# A Hybrid Short-Term Traffic Flow Prediction Model Based on Singular Spectrum Analysis and Kernel Extreme Learning Machine

**DOI:** 10.1371/journal.pone.0161259

**Published:** 2016-08-23

**Authors:** Qiang Shang, Ciyun Lin, Zhaosheng Yang, Qichun Bing, Xiyang Zhou

**Affiliations:** 1 College of Transportation, Jilin University, Changchun 130022, China; 2 State Key Laboratory of Automobile Simulation and Control, Jilin University, Changchun 130022, China; Jiangnan University, CHINA

## Abstract

Short-term traffic flow prediction is one of the most important issues in the field of intelligent transport system (ITS). Because of the uncertainty and nonlinearity, short-term traffic flow prediction is a challenging task. In order to improve the accuracy of short-time traffic flow prediction, a hybrid model (SSA-KELM) is proposed based on singular spectrum analysis (SSA) and kernel extreme learning machine (KELM). SSA is used to filter out the noise of traffic flow time series. Then, the filtered traffic flow data is used to train KELM model, the optimal input form of the proposed model is determined by phase space reconstruction, and parameters of the model are optimized by gravitational search algorithm (GSA). Finally, case validation is carried out using the measured data of an expressway in Xiamen, China. And the SSA-KELM model is compared with several well-known prediction models, including support vector machine, extreme learning machine, and single KLEM model. The experimental results demonstrate that performance of the proposed model is superior to that of the comparison models. Apart from accuracy improvement, the proposed model is more robust.

## 1 Introduction

Short-term traffic flow prediction is one of the most important issues in the field of intelligent transport system (ITS) [1–2]. Many subsystems of ITS such as Advanced Traffic Management System (ATMS) and Advanced Traveler Information Systems (ATIS) can benefit from improved prediction of traffic flow parameters (such as traffic volume, average traffic speed, and average occupancy) in a short-term future.

Many researchers have paid more attention to short-term traffic flow prediction because of its importance. As a result, a large number of relevant methods have been published in the academic literature. In general, these methods are categorized into three types [3–4]: traffic model-based methods, statistical methods and machine learning-based methods. A brief summary of these three types of methods is shown in Table 1.

**Table 1:** A brief summary of the three types of methods.

Types	Advantages	Disadvantages
Traffic model-based methods (macroscopic traffic model, microscopic traffic model)	Well-established theoretical background and good interpretability, consider the effect of various factors (incidents, road works, traffic control measures, etc)	Many parameters and assumptions need to defined in advance, parameter calibration is difficult, small errors of these parameters could lead to large prediction errors
Statistical methods (local linear regression, ARIMA)	comparatively basic structure, easy and practical to be used	Requires a great deal of historical data, gives some restrictive assumptions, difficult to model nonlinear traffic flow
Machine learning-based methods (ANN, SVM)	Gains knowledge from training data, do not need to predefined model structure, good robustness, ability to approximate any degree of complexity of traffic flow	Requires a training procedure and a large number of training data, poor interpretability

1. Traffic model-based methods, which use the traffic flow model (include macroscopic model and microscopic model) to predict the evolving mechanism of traffic flow. Macroscopic model regards the traffic flow as fluid for exploring its evolution mechanism based on fluid dynamics [5–6]. Microscopic model focuses on behavior of single vehicle and the interactions between vehicles, such as lane changing model [7] and car following model [8].
2. Statistical methods, which is implemented by using the historical data to obtain optimal parameters in the fitting process. Typical statistical methods have been proposed and applied for years, such as local linear regression model [9] and Auto-Regressive Integrated Moving Average (ARIMA) [10].
3. Machine learning-based methods, which can effectively capture the nonlinearity relationship between the input and output existed in the data by using intelligent learning algorithm. Such as Artificial Neural Network (ANN) [11–12] and Support Vector Machine (SVM) [13–14].

Reader interested in details of these prediction models can refer to review papers such as Refs. [15–17]. By analyzing the characteristics of the three types of methods, traffic model-based methods and statistical methods often assume several restrictive assumptions, such as a predefined model structure, the normality of residuals and the stationary of the time series, which are seldom satisfied in the case of nonlinear and chaotic traffic flow. However, machine learning-based methods are not restricted by previous assumptions. Particularly, ANN and SVM are more popular because of their mature theoretical basis and the excellent prediction performance.

In recent years, a novel learning algorithm called Extreme Learning Machine (ELM) is proposed for training Single Layer Feed-forward Neural Network (SLFN) [18]. In ELM, input weights and hidden biases are assigned randomly instead of being exhaustively tuned. For this reason, ELM training is fast and saves a lot of computing resources. Moreover, ELM also avoids falling into local minima in the learning process. Therefore, ELM achieves superior performance than traditional SLFN and Back-Propagation (BP) learning algorithm. Because of these advantages of ELM, it has been applied in different fields successfully [19–25]. In order to improve the generalization ability of ELM and reduce time consumption for determining the number of hidden layer nodes, Kernel Extreme Learning Machine (KELM) is developed more recently by replacing the ELM hidden layer with a kernel function [26]. Different with SVM, the kernel function of KELM does not need to satisfy Mercer’s theorem. Thus application of KELM is easier than SVM. In addition, KELM is applied to solve many prediction or classification problems and achieves comparative or superior performance [27–30]. In spite of great success in some scientific fields, the KELM method is not utilized for predicting short-term traffic flow at present. Thus, we have reasons to believe that KELM method has a better application prospect in traffic flow prediction and try to employ KELM method for building a traffic flow prediction model in this paper. At the same time, to obtain the optimal KELM model, it is important to choose a kernel function and determine the model parameters. If the parameters are not selected correctly, the performance of KELM model is greatly reduced. In this paper, Gravitational Search Algorithm (GSA) is introduced to optimize the parameters of KELM model, which is a heuristic optimization method based on the Newtonian gravity law [31]. The GSA has demonstrated potential in parameter determination for nonlinear models [32–34].

Moreover, the input form of KELM model affects its performance and training speed. With the development of chaos theory, recent studies such as Refs. [35–37] have concluded that the nonlinear chaotic phenomena existed in short-term traffic flow time series. Phase Space Reconstruction (PSR) is the basis of chaotic time series analysis, which affects the prediction performance directly. It can map scalar time series to the multi-dimensional phase space and mining the implicit information thoroughly. Therefore, PSR is used to determine the optimal input form of KELM model in this paper. Delay time and embedding dimension are the key parameters for PSR. At present, there are many methods for choosing the two parameters. The methods can be used to calculate delay time including Autocorrelation Function method [38], Mutual Information method [39] and Average Displacement method [40]. The methods can be used to calculate embedding dimension including G-P method [41], False Nearest Neighbors method [42] and Cao method [43]. However, C-C method [44] is different from these methods and can simultaneously estimate delay time and embedding dimension based on statistical results. The advantages of C-C method are small amount of calculation, strong anti-interference ability and easy to use. Thus, we choose C-C method to determine the two parameters for PSR.

The data pre-processing is necessary before training the KELM model. Due to inevitable interference in the process of data acquisition or transmission, the original traffic data contain some noise components and unpredictable components (outliers) which have less useful information and often lead to over-fitting. In pre-processing process, removing noise and outliers of chaotic time series can reduce its complexity and increase its predictability [45]. Spectrum Analysis (SSA) is a relatively new time series analysis technique which combines multivariate statistic, probability theory and signal processing [46]. SSA is suitable for time series with various features and structures, such as stationary and non-stationary, linear and nonlinear time series [47]. It can extract the main features of time series, remove noise and unpredictable components effectively. So far, SSA has received increasing attentions and has been employed in several areas successfully, such as medicine, energy, climatology, and economics [48–51]. Therefore, we choose SSA to filter traffic flow time series in the pre-processing process.

The main objective of this work is to propose a hybrid short-term traffic flow prediction model named SSA-KELM using SSA and KELM. The KELM can achieve a better prediction performance using the filtered traffic flow time series data, because noise components of the original traffic time series have been removed by SSA during the pre-processing process. The novelty of the proposed model is highlighted in the following aspects.

1. The KELM as a relatively novel machine-learning method is first studied for short-term traffic flow prediction.
2. The SSA as an effective data pre-processing method is employed to process the original traffic flow series.
3. In view of the chaotic characteristics of traffic flow, PSR is performed to determine the optimal input form of the KELM.
4. The parameters of KELM are tuned by gravitational search algorithm (GSA) to achieve a better prediction performance.
5. Non-parametric statistical tests are used for multiple comparisons of different prediction models on multiple data sets.

The rest of this paper is organized as follows. In Section 2, SSA method and KELM model are described briefly, and the hybrid traffic flow prediction model (SSA-KELM) is introduced. In Section 3, empirical analysis is performed, the prediction results of several different prediction models are given and discussed. In Section 4, conclusions and recommendations for future study are presented.

## 2. Methodology

In this setction, the basic methods related to the SSA-KELM model are briefly introduced, including Singular Spectrum Analysis (SSA), Kernel Extreme Learning Machine (KELM), Phase Space Reconstruction (PSR) and Gravitational Search Algorithm (GSA). Then the main steps of SSA-KELM model are given. In the SSA-KELM model, KELM is the basic prediction method, SSA is used to filter the noise components from the original traffic data, PSR is used to determine the optimal input form of KELM and the GSA is used to optimized the paramenters of the KELM.

### 2.1 Singular spectrum analysis (SSA)

SSA performs four steps including embedding, singular value decomposition, grouping and diagonal averaging. The first two steps are usually named decomposition of time series, while the third step and fourth step called reconstruction of time series. A brief review of SSA is as follows (more information can be obtained in Ref. [52]).

Step 1: Embedding. This step is to transform the original series into a sequence of multi-dimensional vector. For one dimensional series vector with length *N* as **X** = (*x*_1_, ⋯, *x*_*N*_). Given a window length *L*(1 < *L* < *N*), the initial series is mapped into *K* lagged vectors:

$$X_{i}={\left(x_{i},x_{i+1},\cdots ,x_{i+L-1}\right)}^{T},i=1,2,\cdots ,K=N-L+1$$ (1)

Then, the trajectory matrix is expressed as Eq (2). The matrix **T** is a Hankel matrix with size of *L* × *K*, which has equal elements *x*_*ij*_ along the anti-diagonals where *i* + *j* = const,

$$T=\left[X_{1},X_{2},\cdots ,X_{K}\right]=\left[\begin{array}{cccc} x_{1} & x_{2} & \cdots & x_{K}\\ x_{2} & x_{3} & \cdots & x_{K+1}\\ \cdots & \cdots & \cdots & \cdots \\ x_{L} & x_{L+1} & \cdots & x_{N} \end{array}\right]$$ (2)

Step 2: Singular Value Decomposition (SVD). Let **S** = **TT**^*T*^, the eigenvalues of **S** are calculated and arranged in decreasing order, denoted as *λ*_1_ ≥ ⋯ ≥*λ*_*L*_ ≥ 0. The eigenvectors of matrix **S** corresponding to these eigenvalues are denoted as **U**_1_, ⋯ **U**_*L*_. Then the SVD of trajectory matrix **X** is defined as Eq (3):

$$T=T_{1}+\cdots +T_{d}$$ (3)

where *d* is the rank of **T**, $T_{i}=\sqrt{\lambda_{i}}U_{i}V_{i}^{T}\left(i=1,\cdots ,d\right)$ are elementary matrix (rank is 1), $V_{i}=T^{T}U_{i}/\sqrt{\lambda_{i}}$ are principal components of matrix **T**. The collection $\left(\sqrt{\lambda_{i}},U_{i},V_{i}\right)$ is termed as *i*th eigen triple of SVD. The contribution of **T**_*i*_ can be measured by ratio of eigenvalues and given by Eq (4),

$$\eta_{i}=\lambda_{i}/\overset{d}{\underset{i=1}{\sum }}\lambda_{i}$$ (4)

Step 3: Grouping. Indices set {1, ⋯, *d*} is divided into *m* disjointed subsets *I*_1_, ⋯, *I*_*m*_. Let *I* = {*i*_1_, ⋯, *i*_*p*_} and the resultant trajectory matrix **T**_*I*_ corresponding to the group *I* is defined as $T_{I}=T_{i_{1}}+\cdots +T_{i_{p}}$. The resultant trajectory matrices are calculated for every group *I* = *I*_1_, ⋯, *I*_*m*_ and the expansion of Eq (3) leads to the decomposition as follows.

$$T=T_{I_{1}}+\cdots +T_{I_{m}}$$ (5)

Step 4: Diagonal averaging: the grouped decomposition in Eq (5) is transformed a new series with length *N*. Let **Y**_1_ = (*y*_1_, ⋯, *y*_*N*_) be the transformed one dimensional series of $T_{I_{1}}$, elements of **Y**_1_ is calculated by Eq (6).

$$y_{k}=\left\{ \begin{array}{l} \frac{1}{k}\sum_{j=1}^{k}y_{j,k-j+1}^{*},1\leq k\leq L^{*}\\ \frac{1}{L^{*}}\sum_{j=1}^{L^{*}}y_{j,k-j+1}^{*},L^{*}\leq k\leq K^{*}\\ \frac{1}{N-k+1}\sum_{j=k-K+1}^{N-K^{*}+1}y_{j,k-j+1}^{*},K^{*}\leq k\leq N \end{array}\right.$$ (6)

where L* = min(*L*, *K*), K* = max(*L*, *K*); if *L* < *K*, $y_{j,k-j+1}^{*}=y_{j,k-j+1}$ and if *L* ≥ *K*, $y_{j,k-j+1}^{*}=y_{k-j+1,j}$. Thus the original series **X** = (*x*_1_, ⋯, *x*_*N*_) is decomposed into *m* series:

$$Y=Y_{1}+\cdots +Y_{m}$$ (7)

### 2.2 Kernel extreme learning machine (KELM)

A brief description of ELM and KELM is given in this section. Interested readers can refer to Refs. [18, 26] for more details. The output function of ELM for generalized SLFN is

$$f_{L}\left(x\right)=\overset{L}{\underset{i=1}{\sum }}\beta_{i}h_{i}\left(x_{j}\right)=\overset{L}{\underset{i=1}{\sum }}\beta_{i}g_{i}\left(w_{i},b_{i},x_{j}\right)=h\left(x\right)\beta ,j=1,\cdots ,N$$ (8)

where *g*_*i*_(·) denotes the output of the *i*-th hidden node with respect to the input *x*, i.e., activation function, such as “Sigmoid” function. *w*_*i*_ ∈ *R*^*n*^ is the weight vector connecting the *i*th hidden layer neuron and the input layer neurons, *b*_*i*_ the bias of the *i*th hidden layer neuron, *β*_*i*_ ∈ *R* is the weight connecting the *i*th hidden neuron and the output neuron, and *f*_*L*_(*x*) is the output of the SLFN. *w*_*i*_ and *b*_*i*_ are randomly assigned before learning.

If SLFN can approximate theses *N* samples with zero error, there exists *β*_*i*_, *w*_*i*_ and *b*_*i*_ such that

$$\overset{L}{\underset{i=1}{\sum }}\beta_{i}g\left(w_{i},b_{i},x_{j}\right)=t_{j},j=1,2,\ldots ,N$$ (9)

Thus Eq (9) is expressed compactly as

Hβ=T (10)

Where

$$H=\left(\begin{array}{c} h\left(x_{1}\right)\\ \vdots \\ h\left(x_{N}\right) \end{array}\right)={\left[\begin{array}{ccc} h(w_{1},b_{1},x_{1}) & \cdots & h(w_{L},b_{L},x_{1})\\ \vdots & \ddots & \vdots \\ h(w_{1},b_{1},x_{N}) & \cdots & h(w_{L},b_{L},x_{N}) \end{array}\right]}_{N\times L}$$ (11)

$$\beta ={\left(\begin{array}{c} \beta_{1}^{T}\\ \vdots \\ \beta_{L}^{T} \end{array}\right)}_{L\times m}\text{ and }T={\left(\begin{array}{c} t_{1}^{T}\\ \vdots \\ t_{N}^{T} \end{array}\right)}_{N\times m}$$ (12)

Where **H** is the hidden layer output matrix, **β** is output weight matrix, and **T** is the target matrix. In the ELM, **β** is the only parameter that needs to be calculated and can be easily achieved by Least Squares Estimate (LSE)

$$\beta =H^{†}T$$ (13)

Where **H**^†^ is Moore-Penrose generalized inverse of matrix **H**. one of the methods for calculating **H**^†^ is the orthogonal projection method:

$$H^{†}=H^{T}{\left(HH^{T}\right)}^{\text{-1}}$$ (14)

According to the ridge regression theory, the diagonal of **HH**^*T*^ can be add a positive value for regularization, then we have

$$f\left(x\right)=h\left(x\right)\beta =h\left(x\right)H^{T}{\left(\frac{I}{C}HH^{T}\right)}^{-1}T$$ (15)

Where **I** is a unit matrix, *C* is penalty parameter. When the hidden feature mapping function *h*(*x*) is unknown, a kernel matrix for ELM is used according to the following equation:

$$\Omega_{\text{ELM}}=HH^{T}:\Omega_{{\text{ELM}}_{i\text{,}j}}=h\left(x_{i}\right)\cdot h\left(x_{j}\right)=K\left(x_{i},x_{j}\right)$$ (16)

where *K*(*x*_*i*_, *x*_*j*_) is a kernel function. Thus, the output function of ELM is denoted compactly as

$$f\left(x\right)={\left[\begin{array}{c} K\left(x,x_{1}\right)\\ \vdots \\ K\left(x,x_{N}\right) \end{array}\right]}^{T}{\left(\frac{I}{C}+\Omega_{\text{ELM}}\right)}^{-1}T$$ (17)

Because of the application of kernel function in ELM, this novel learning algorithm is named KELM. In KELM, weight vector *w*_*i*_, bias *b*_*i*_, the feature mapping function *h*(*x*) and the number of hidden layer neurons *L* are not taken into consideration. Instead, KELM only focuses on the kernel functions *K*(*x*_*i*_, *x*_*j*_) and the training data set. Many kernel functions can be used for KELM, such as linear, polynomial and Gaussian radial basis function (RBF). We choose the Gaussian RBF kernel *K*(*u*, *v*) = exp(−‖*u* − *v*‖^2^ / 2*σ*^2^) to construct KELM model because of its superior performance [26, 30]. In the KELM with Gaussian RBF kernel, the are two key parameters (penalty parameter *C* and kernel parameter *σ*) need to be determined.

### 2.3 Phase space reconstruction (PSR)

According to Takens’ Embedding Theorem [53], as enough delayed coordinates are used, scalar time series is sufficient to reconstruct the dynamic of the underlying systems. For a time series {*x*(*i*), *i* = 1, 2, ⋯, *N*}, the phase space can be reconstructed according to

X(i)={x(i),x(i+τ),⋯,x[i+(m−1)τ]} (18)

Where *τ* is delay time, *m* is embedding dimension. According to phase space reconstruction. Input of the model is [*x*_*i*_, *x*_*i+τ*_, ⋯, *x*_*i*+(*m*−1)*τ*_] and output of the model is *x*_*i*+(*m*−1)*τ*+1_.

The C-C method is used to determine delay time *τ* the embedding dimension *m*. According to Ref. [39], the basic principle of C-C method is as follows.

The correlation integral is defined as

$$C\left(m,N,r,t\right)=\frac{2}{M\left(M-1\right)}\underset{1\leq i\leq j\leq M}{\sum }H\left(r-d_{ij}\right)$$ (19)

where *r* is the search radius, *M* = *N*–(*m*– 1)*τ* is the number of embedded points in *m*-dimensional space, *N* is the data number of the time series. *d*_*ij*_ = ‖*X*_*i*_−*X*_*j*_‖ is Euclidean distance between two points, *H*(*z*) is a Heaviside function

$$H\left(z\right)=\left\{ \begin{array}{l} 1\;\left(z>0\right)\\ 0\;\left(z\leq 0\right) \end{array}\right.$$ (20)

The correlation integral is a cumulative distribution function and denotes the probability of distance that between any pairs of points in the phase space is not greater than *r*.

The time series {*x*(*i*), *i* = 1, 2, ⋯, *N*} is divided into *t* disjoint time series:

$$\begin{array}{l} \left\{x\left(1\right),x\left(t+1\right),x\left(2t+1\right),\cdots \right\}\\ \left\{x\left(2\right),x\left(t+2\right),x\left(2t+2\right),\cdots \right\}\\ \;\;\;\;\;\;\vdots \\ \left\{x\left(3\right),x\left(t+3\right),x\left(2t+3\right),\cdots \right\} \end{array}$$ (21)

The test statistics is

$$S\left(m,N,r,t\right)=\overset{t}{\underset{l=1}{\sum }}\frac{1}{t}\left[C_{l}(M,\frac{N}{r},r,t)-C_{l}^{m}\left(1,\frac{N}{r},r,t\right)\right]$$ (22)

As *N* → ∞, the Eq (22) can be expressed as

$$S\left(m,r,t\right)=\overset{t}{\underset{l=1}{\sum }}\frac{1}{t}\left[C_{l}(M,r,t)-C_{l}^{m}\left(1,r,t\right)\right]$$ (23)

The maximum deviation Δ*S*(*m*, *t*) of *S*(*m*, *r*, *t*) ~ *t* with *r* is defined as

$$\Delta S\left(m,t\right)=\left\{\text{max}S\left(m,r_{i},t\right)-\text{min}S\left(m,r_{j},t\right)\right\}$$ (24)

According to the BDS statistical results obtained by Brock et al. [54], *m* = 2, 3, 4, 5, *r*_*i*_ = *iσ* / 2 and *i* = 1, 2, 3, 4 is selected in general, where *σ* is standard deviation of time seires. Calculate the following variables,

$$\bar{S}(t)=\frac{1}{16}\overset{5}{\underset{m=2}{\sum }}\overset{4}{\underset{j=1}{\sum }}S(m,r_{j},t)$$ (25)

$$\Delta \bar{S}(t)=\frac{1}{4}\overset{5}{\underset{m=2}{\sum }}\Delta S\left(m,t\right)$$ (26)

$$Scor(t)=\Delta \bar{S}(t)+\left|\bar{S}(t)\right|$$ (27)

where $\bar{S}(t)$ is the mean of *S*(*m*, *r*, *t*) for all subsequence. Let *t* equal or be smaller than 200, the first local minimum point of $\Delta \bar{S}(t)∼t$ is the delay time *τ*. The global minimum point of *Scor*(*t*) ~ *t* is the delay time window *τ*_*w*_. The embedding dimension *m* is calculated according to *τ*_*w*_ = (*m* − 1)*τ*.

### 2.4 Gravitational search algorithm (GSA)

In GSA, a set of agents has been given to find optimum solution based on Newtonian gravity law. Assume agents with number *N* in the dimension of *n*, and the position of *i*th agent is defined as:

$$X_{i}=\left(x_{i}^{1},\cdots ,x_{i}^{d},\cdots ,x_{i}^{n}\right),i=1,2,\cdots ,N$$ (28)

Where $x_{i}^{d}$ is the position of *i*th agent in the *d*th dimension. The main steps of GSA are as follows:

Step 1: Initialize velocity and positon of each agent.

Step 2: Evaluate the fitness of each agent.

Step 3: Update gravitational constant *G*(*t*) according to the following equation:

$$G\left(t\right)=G_{0}*e^{-\alpha /T}$$ (29)

where *G*_0_ is the initial gravitational constant, *α* is decay rate (some arbitrary constant) and *T* is the maximum number of iterations.

Step 4: Calculate the gravitational (inertia) mass of each agent using the following equation:

$$\left\{ \begin{array}{l} m_{i}\left(t\right)=\frac{fit_{i}\left(t\right)-worst\left(t\right)}{best\left(t\right)-worst\left(t\right)}\\ M_{i}\left(t\right)=\frac{m_{i}\left(t\right)}{\sum_{j=1}^{N}m_{j}\left(t\right)} \end{array}\right.$$ (30)

Where *M*_*i*_(*t*) and *fit*_*i*_(*t*) are the mass and the fitness value of the agent *i* at time *t*, respectively. *worst*(*t*) and *best*(*t*) are the worst fitness value of agent and the best fitness value of agent, respectively.

Step 5: Calculate the resultant force of agents according to the law of universal gravitation.

$$F_{ij}^{d}\left(t\right)=G\left(t\right)\frac{M_{i}\left(t\right)\cdot M_{j}\left(t\right)}{R_{ij}\left(t\right)+\varepsilon }\cdot \left(x_{i}^{d}\left(t\right)-x_{j}^{d}\left(t\right)\right)$$ (31)

where $F_{ij}^{d}\left(t\right)$ is the gravitation between the agent *i* in the *d* dimension and agent *j* at time *t*; *M*_*i*_(*t*) and *M*_*j*_(*t*) are the passive gravitational mass of agent *i* and agent *j* respectively; *ε* is the small constant for avoiding the divisor equal to zero; $x_{i}^{d}\left(t\right)$ is the position of agent *i* in *d* dimension and $x_{j}^{d}\left(t\right)$ is the position of agent *j* at time *t*; *R*_*ij*_(*t*) = ‖*x*_*i*_(*t*), *x*_*j*_(*t*)‖_2_ is the Euclidean distance between agent *i* and agent *j*.

Step 6: Calculate accelerated velocity of agent. Accelerated velocity $a_{i}^{d}\left(t\right)$ at time *t* in *d* dimension is expressed as follows.

$$a_{i}^{d}\left(t\right)=\frac{\sum_{i=1,j\neq 1}^{N}rand\cdot F_{ij}^{d}\left(t\right)}{M_{i}\left(t\right)}$$ (32)

where *rand* is a random number in the interval [0, 1].

Step 7: Update the positon and velocity of each agent according to Eqs (33) and (34) respectively.

$$v_{i}^{d}\left(t+1\right)=rand\cdot v_{i}^{d}\left(t\right)+a_{i}^{d}\left(t\right)$$ (33)

$$x_{i}^{d}\left(t+1\right)=x_{i}^{d}\left(t\right)+v_{i}^{d}\left(t+1\right)$$ (34)

Step 8: If the stopping criterion is satisfied, output optimal parameters, else proceed to Step 2.

### 2.5 SSA-KELM model

**Fig 1:**
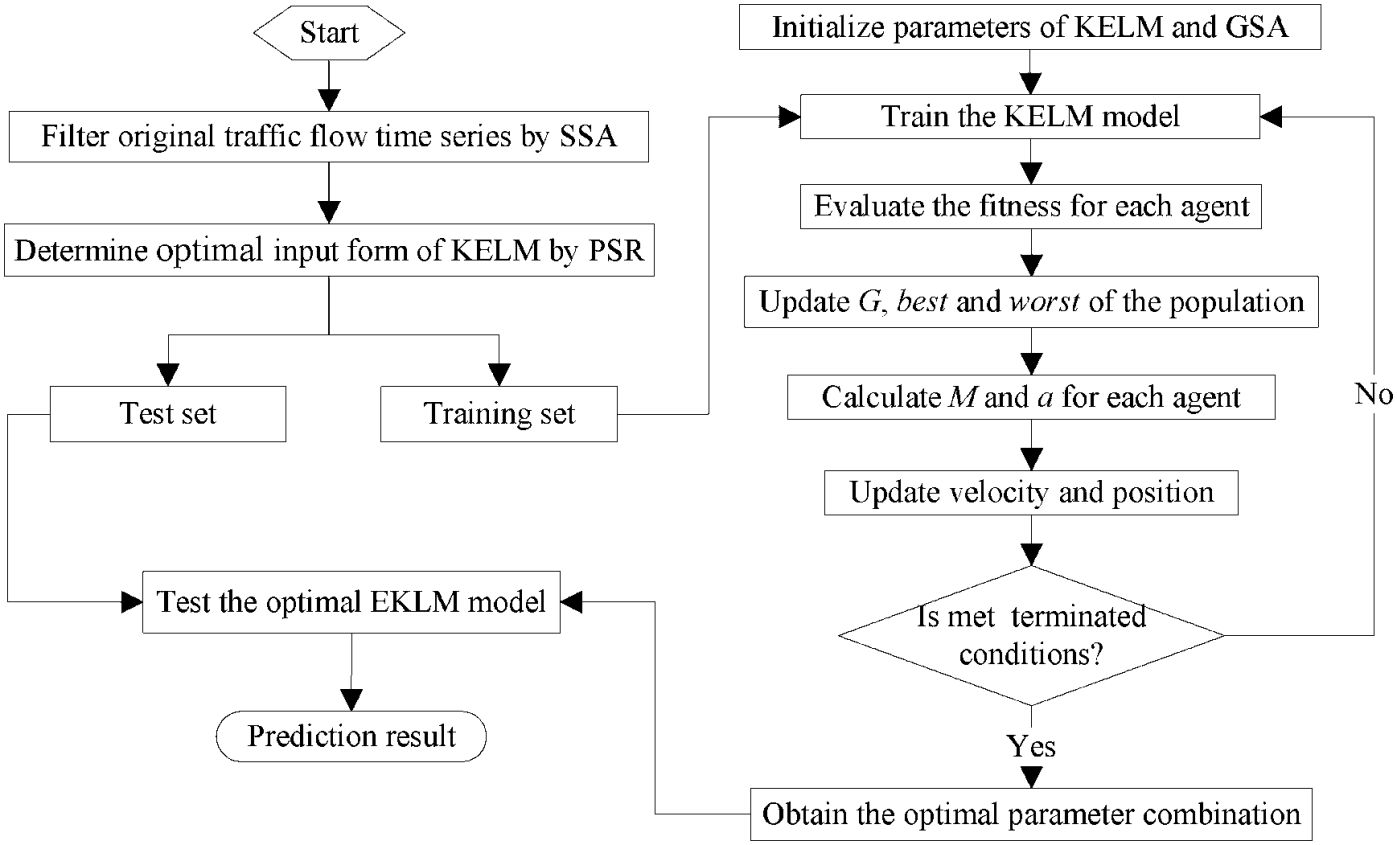
The flowchart of SSA-KELM Model.

The overall flowchart of SSA-KELM model is illustrated as Fig 1. And the main steps of SSA-KELM model are as follows.

Step 1: The original traffic flow time series is filtered by SSA and get a reconstruction time series without nosie components.

Step 2: The optimal input form of the KELM is determined by PSR. The filtered traffic flow time series is used to train the KELM model, while the original traffic flow time series is used to test the KELM model.

Step 3: The parameters of KELM model are optimized by GSA.

(3.1) Initialize the parameters of the KELM model and GSA.
(3.2) Train the KELM model and evaluate the fitness value of each agent.
(3.3) Update *G*(*t*), *best*(*t*) and *worst*(*t*) of the population.
(3.4) Calculate *M*_*i*_(*t*) and $a_{i}^{d}\left(t\right)$ for each agent.
(3.5) Update the positon and velocity of each agent.
(3.6) Judge whether the terminated conditions are reached (usually the default calculation accuracy or iterations). If reached, continue to step 3.7; otherwise, go to step 3.2 and continue to search.
(3.7) Output the optimal parameters of KELM model.

## 3 Empirical research

In this section, the measured data is used to validate the performance of SSA-KELM model. First, a detailed description of the data source is given. Then, the traffic data is used to illustrate how to construct and optimize the SSA-KELM model. Finally, the performance of the SSA-KELM model is compared to that of several prediction models on multiple data sets. Besides the traditional statistical indices are employed to evaluate the performances of all the prediction models, non-parametric tests are used to analyze the results of the contrast experiment.

### 3.1 Data Source

**Fig 2:**
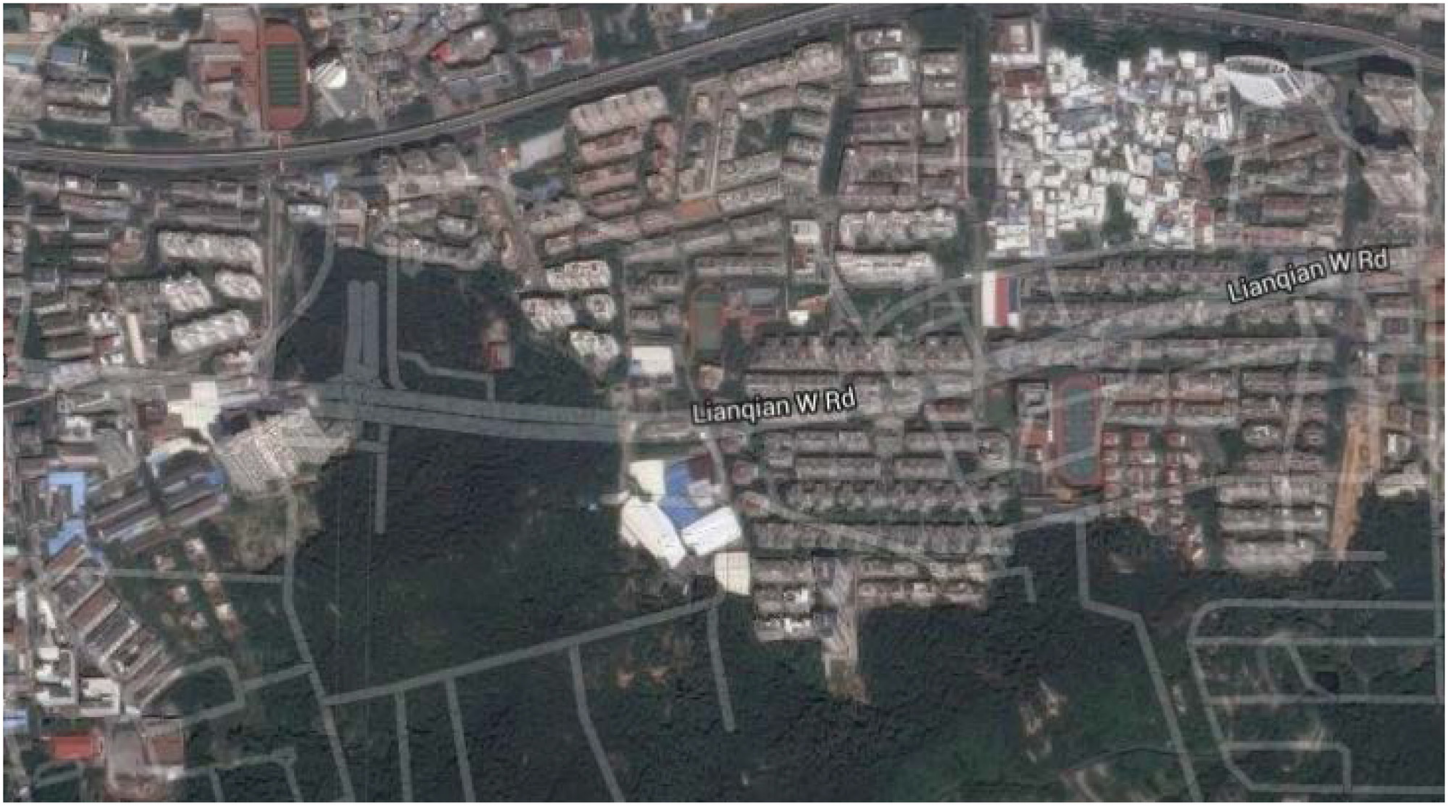
The two detectors’ locations on the Lianqian W Rd.

**Fig 3:**
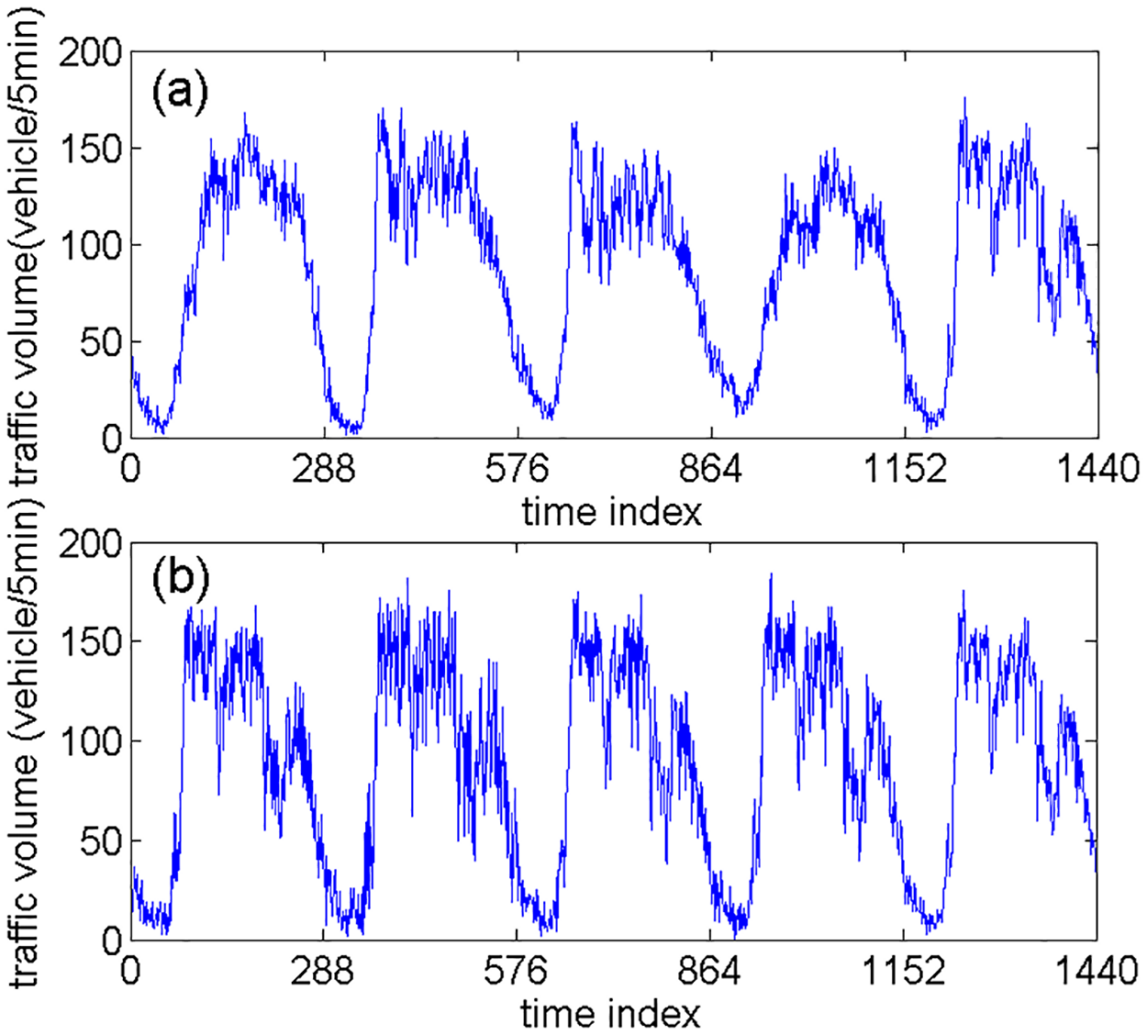
The traffic volume data in five consecutive working days: (a) DC00004965 and (b) DC00004966.

All the experimental traffic volume data is obtained from two loop detectors (No. DC00004965 and No. DC00004966) installed on an expressway named Lianqian W Rd in Xiamen, China. The two detectors’ locations are approximately shown in Fig 2, where No. DC00004965 detector locates in the westbound direction while No. DC00004966 detector locates in the eastbound direction. The traffic data is collected every 5 min in five consecutive working days (January 5, 2015 to January 9, 2015). Although average speed is also available, only traffic volume is considered in this study. The original traffic volume data is shown in Fig 3. The traffic volume data of the first four days is used to construct the SSA-KELM model, while the traffic volume data of the fifth day is used to test the KELM model. In the following several subsections, traffic volume data from No. DC00004966 detector is used as an example to illustrate how to build and optimize the SSA-KELM model in detail, while traffic volume data from the two detectors is all used to evaluate the performance of the SSA-KELM model.

### 3.2 SSA decomposition and reconstruction

**Fig 4:**
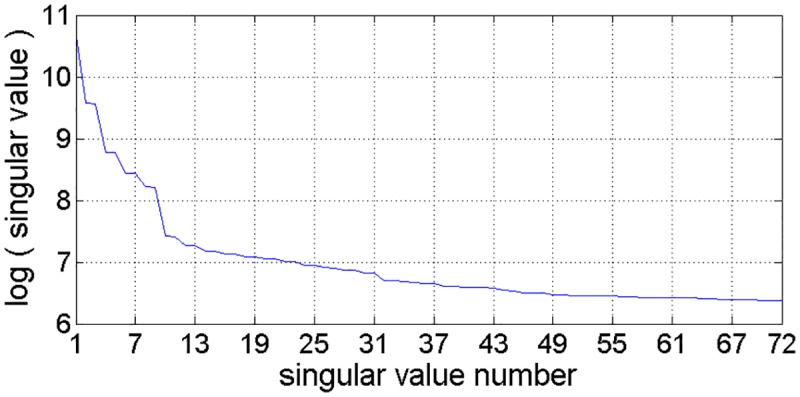
Singular values curve (in descending order).

As mentioned in subsection 2.1, the window length *L* is the only parameter in the decomposition process. If the time series has a periodic component, the window length is taken proportional to that period to get better separability [55]. Therefore, *L* = 288 is assumed here, which corresponds to daily variations of traffic volume time series. This window length results in 288 Eigen triples. Each Eigen triples corresponds to a singular value. Fig 4 depicts the curve of leading 72 singular values in descending order (take the base-10 logarithm). As shown in Fig 4, the leading 31 singular values decrease rapidly while the remaining singular values decrease slowly, which means that the remaining eigen triples correspond to the noise components and can be ignored in the reconstruction process. Moreover, the singular value pairs with same or similar singular value, such as {2, 3}, {4, 5}, {6, 7} and …, which correspond to oscillatory components.

**Fig 5:**
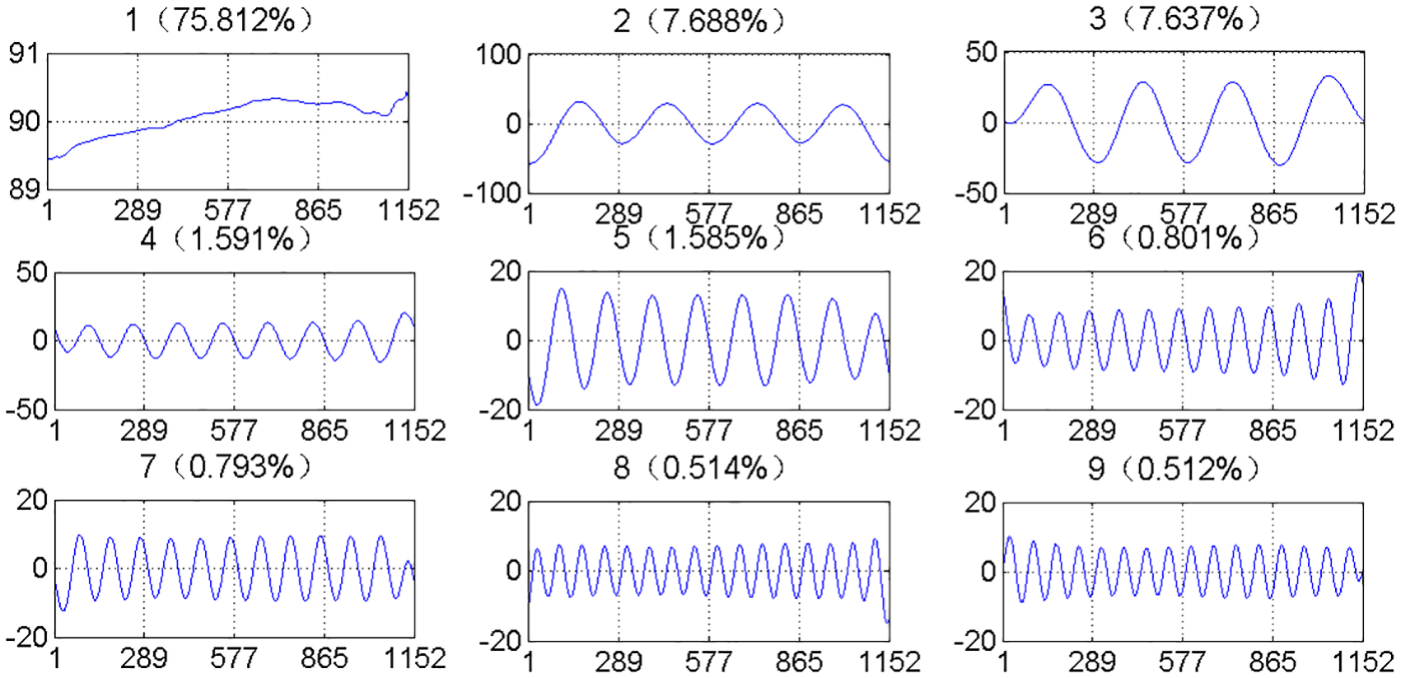
The reconstruction series and contribution ratio corresponding to the leading 9 Eigen triples.

Fig 5 shows the reconstruction traffic volume time series and contribution ratio corresponding to the leading 9 Eigen triples. The first Eigen triple almost represents the whole trend of the original traffic volume series, which has a share (contribution) of 75.812% of original traffic volume series. Every oscillatory component produces two Eigen triples with close singular values, such as {2, 3}, {4, 5}, {6, 7}, {8, 9}, which have a close frequency and a close contribution ratio.

**Fig 6:**
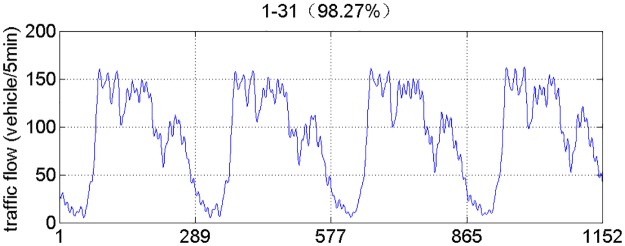
The reconstruction series.

**Fig 7:**
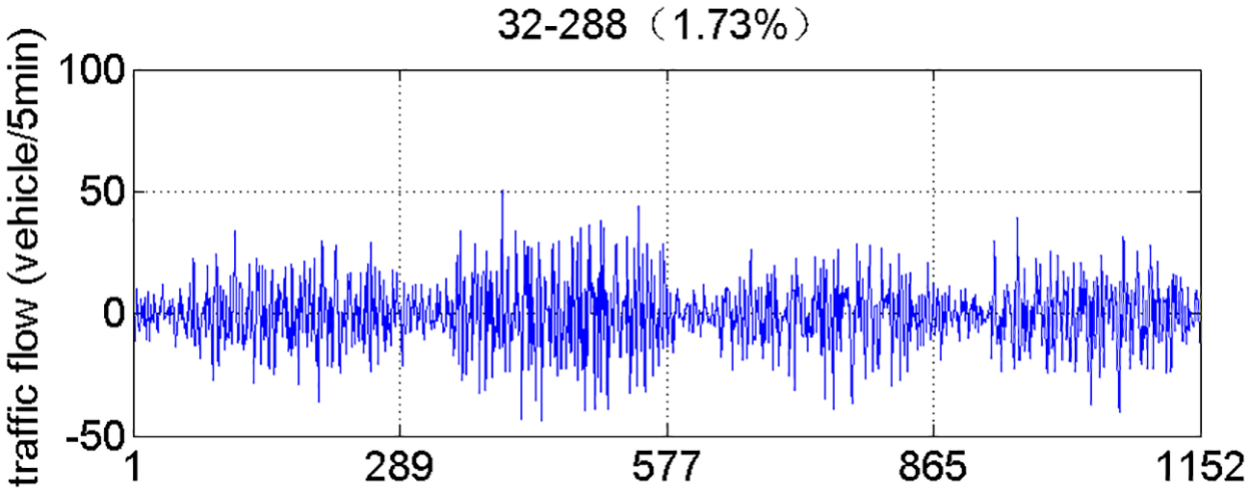
The noise (residual) series.

According to the above analysis, the traffic volume series is reconstructed by using the leading 31 Eigen triples. Fig 6 shows the reconstruction series with a share of 98.27% of original traffic volume series. Fig 7 shows the noise (residual) series with a share of 1.73% of original traffic volume series. As shown in Fig 7, we can see it clearly that the reconstruction series has a satisfactory residual.

### 3.3 Determination of the optimal input form

**Fig 8:**
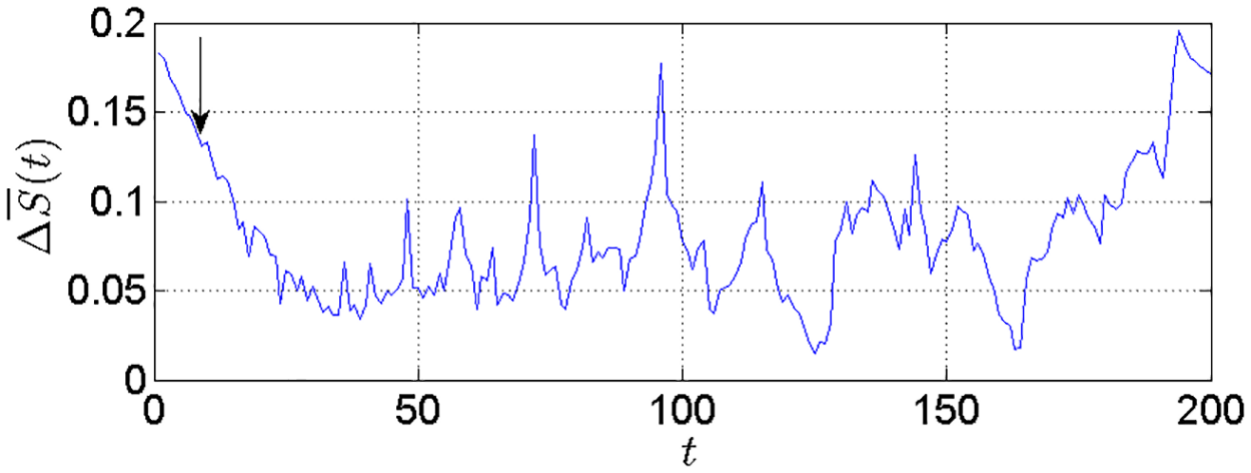
The $\Delta \bar{S}\left(t\right)∼t$ curve.

**Fig 9:**
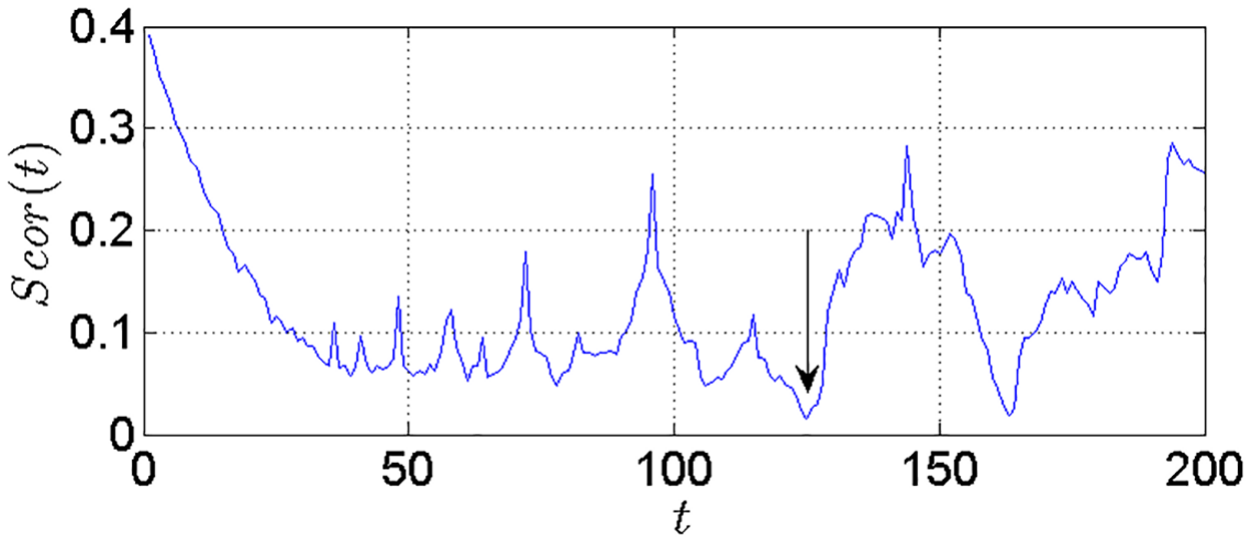
The *Scor*(*t*) ~ *t* curve.

The C-C method is used to implement phase space reconstruction for determining the optimal input form of the proposed model. Fig 8 shows the $\Delta \bar{S}\left(t\right)∼t$ curve. The variable *t* corresponding to the first local minimum value of $\Delta \bar{S}(t)$ is the time delay *τ* and we can get *τ* = 9. Fig 9 shows *Scor*(*t*) ~ *t* curve. The variable *t* corresponding to the global minimum value of *Scor*(*t*) is the embedding window width *τ*_*w*_ of the time series and we can get *τ*_*w*_ = 125. Thus, the embedding dimension *m* = 15 according to *τ*_*w*_ = (*m* − 1)*τ*. Based on phase space reconstruction, input and output of the training set are as follows.

$$\text{input}=\left[\begin{array}{ccccc} x_{1} & x_{1+9} & x_{1+2\times 9} & \cdots & x_{1+(15-1)\times 9}\\ x_{2} & x_{2+9} & x_{2+2\times 9} & \cdots & x_{2+(15-1)\times 9}\\ x_{3} & x_{3+9} & x_{3+2\times 9} & \cdots & x_{3+(15-1)\times 9}\\ \vdots & \vdots & \vdots & \ddots & \vdots \\ x_{1025} & x_{1025+9} & x_{1025+2\times 9} & \cdots & x_{1025+(15-1)\times 9} \end{array}\right]$$

$$\text{output}=\left[\begin{array}{c} x_{1+(15-1)\times 9+1}\\ x_{2+(15-1)\times 9+1}\\ x_{3+(15-1)\times 9+1}\\ \vdots \\ x_{1025+(15-1)\times 9+1} \end{array}\right]$$

### 3.4 Parameter Optimization

GSA is employed to optimize the two parameters of the KELM model. In order to ensure the algorithm efficiency, the mean absolute percentage error is taken as fitness function. Then the fitness function is calculated as follows.

$$fitness=\frac{1}{n}\sum_{i=1}^{n}\left|\frac{y_{i}-{\tilde{y}}_{i}}{y_{i}}\right|$$ (35)

Where *y*_*i*_ is the real value in time interval *i*, ${\tilde{y}}_{i}$ is the prediction value for time interval *i* and *n* is the total number in the time series.

The specific parameters of GSA are as follows: population size is set to 20, the initial gravitational constant *G*_0_ = 100, the attenuation rate *α* = 10, the maximum number of iteration is set to 100, the value range of parameter *C* is set to [0.1, 1000] and the value range of kernel parameter *σ* is set to [0.01, 100].

**Fig 10:**
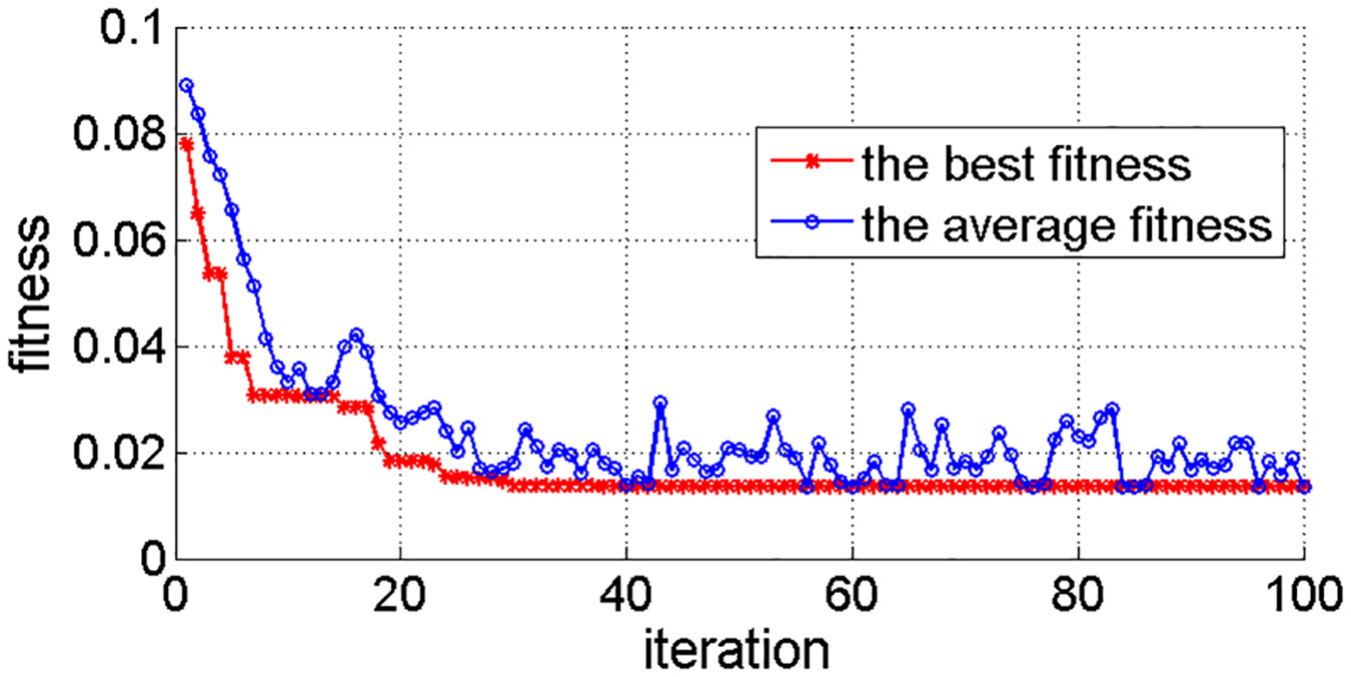
Fitness curves of GSA.

In the process of parameter optimization, the *k*-cross validation method [56] is used to train the model. Because this method can make full use of the information in the sample and avoid over-fitting and under-fitting. In other words, it can improve the generalization ability of the model under the premise of ensuring good prediction accuracy. In *k*-cross validation, the training data set is randomly divided into *k* subsets. The *k* − 1 subsets are used as training set for building the model and the *k*th subset is used as a validation set for verifying model performance. Each subset is used as a validation set and the verifying is repeated *k* times in total. The average value of the results of *k* times verifying is used to evaluate the model performance. Fig 10 shows the fitness curves (include the best fitness and the average fitness). As shown in Fig 10, with the increase of the number of iterations, the fitness curve is gradually convergent. When the number of iterations is 96, both the best fitness and average fitness are 0.0135 (MAPE = 1.35%). And the corresponding optimal parameter combination of the model is *C* = 7.28 and *σ* = 0.15.

### 3.5 Performance Evaluation Index

In order to evaluate the performance of the proposed model, two statistical indices are utilized to measure the prediction accuracy. These indices are the MAE (mean absolute error) and MAPE (mean absolute percent error). The smaller values of MAE and MAPE, the more accurate prediction results are. The calculation formulas for the MAE and MAPE are as follows:

$$MAE=\frac{1}{n}\sum_{i=1}^{n}\left|y_{i}-{\tilde{y}}_{i}\right|$$ (36)

$$MAPE=\frac{1}{n}\sum_{i=1}^{n}\left|\frac{y_{i}-{\tilde{y}}_{i}}{y_{i}}\right|$$ (37)

where *y*_*i*_ is the real value in time interval *i*, ${\tilde{y}}_{i}$ is the prediction value for time interval *i* and *n* is the total number of the time series.

### 3.6 Model Performance and Analysis

**Fig 11:**
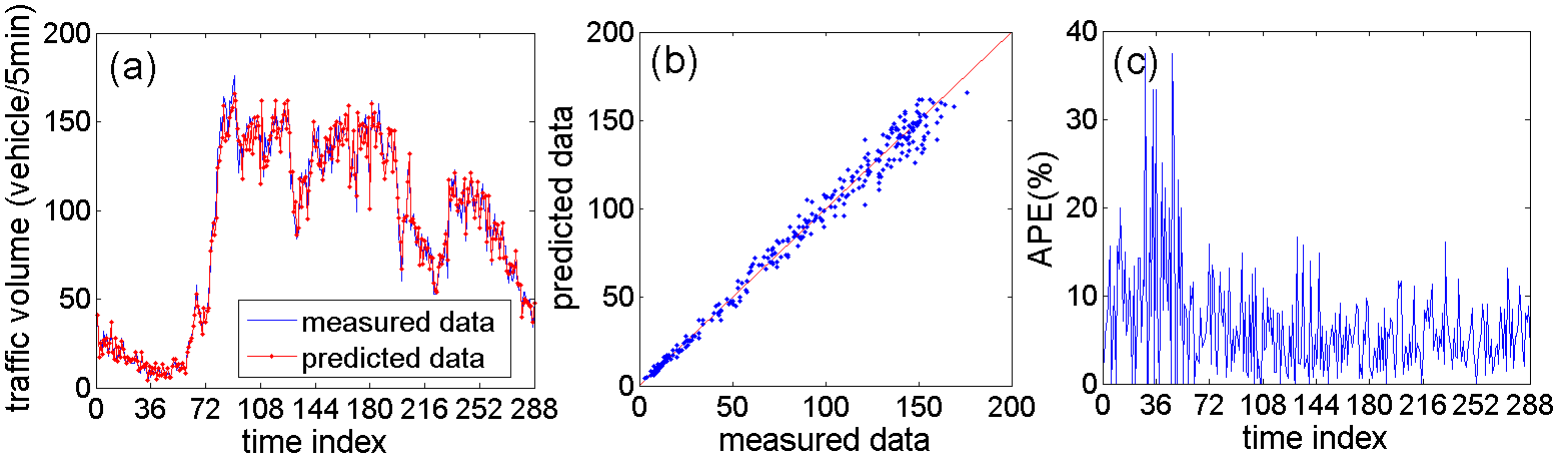
The prediction performance of the proposed method based on NO. DC00004965 detector’s data: (a) Curves of predicted data and measured data; (b) Scatterplot of predicted data and measured data; (c) Absolute percent error of predicted data.

**Fig 12:**
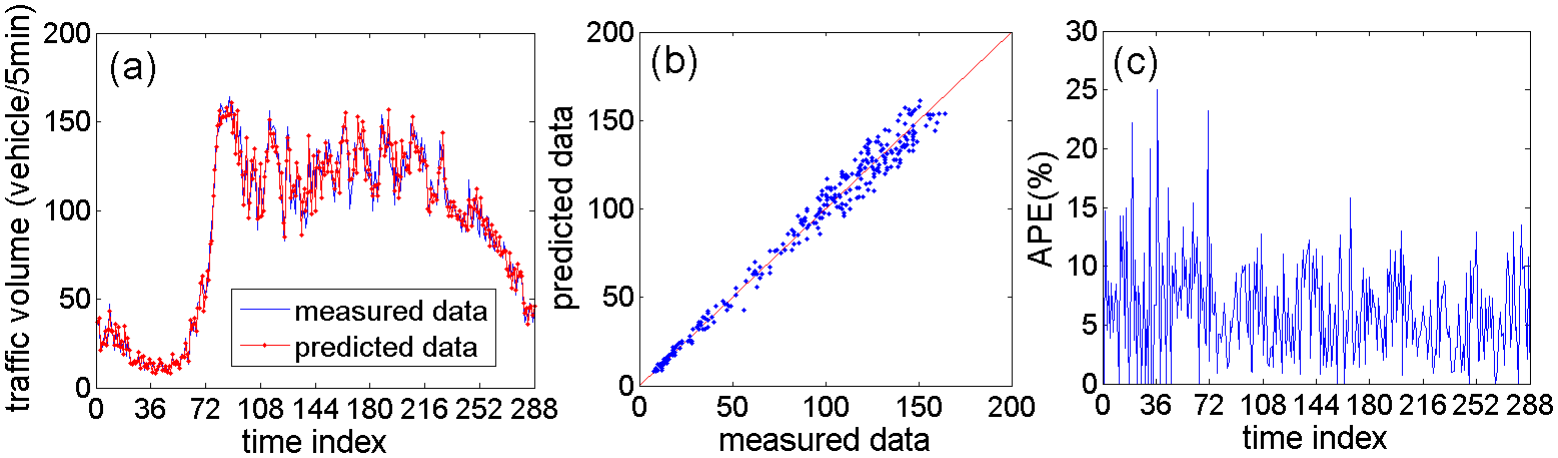
The prediction performance of the proposed method based on NO. DC00004966 detector’s data: (a) Curves of predicted data and measured data; (b) Scatterplot of predicted data and measured data; (c) Absolute percent error of predicted data.

Traffic volume data from the fifth day is used as test set to evaluate the performance of the proposed model. For illustrating the model performance intuitively, Figs 11 and 12 show prediction results of the proposed model using NO. DC00004965 detector’s data and NO. DC00004966 detector’s data, respectively. Figs 11(a) and 12(a) present the curves of prediction data and measured data. The prediction curves can fit the measured curves well. Figs 11(b) and 12(b) present the scatterplots between the predicted data and the measured data. It is clear that these scatter points distribute near the measured line (the red line) without large deviation. Figs 11(c) and 12(c) show APE (absolute percent error) of the predicted data. The APE are mostly within 15%. However, the APE from 24 to 48 (correspond to 2:00–4:00) is larger, and the reason is that the actual traffic volume data is too small during that period.

To illustrate the superiority of the SSA-KELM model, contrast experiment is carried out. In this paper, the related models for comparsion are as follows: the single model (KELM), the hybrid model (SSA-SVM) based on SSA and SVM, and the hybrid model (SSA-ELM) based on SSA and ELM. The optimal input forms of all models are determined by PSR, and the training sets of SSA-SVM model and SSA-ELM model are the same as the SSA-KELM model. However, the original traffic volume data without being filtered by SSA is used to train the KELM model. The parameters of the related models are optimized by GSA, shown in Table 2.

**Table 2:** Parameters of the related models.

Model	parameters
**KELM**	penalty parameter is 18.5
	RBF kernel parameter is 0.41
**SSA-SVM**	penalty parameter is 53.6
	RBF kernel parameter is 0.02
**SSA-ELM**	the number of hidden layer neurons is 27

Besides related models (SSA-SVM, KELM and SSA-ELM) mentioned above, two state-of-the-art traffic flow prediction models are used for comparison, that are Hybrid Particle Swarm Optimization Support Vector Regression (HPSO-SVR) model [57] and Long Short-Term Memory Neural Network (LSTM-NN) model [58]. In the contrast experiment, we implement the two state-of-the-art models according to detail steps of the corresponding references.

**Fig 13:**
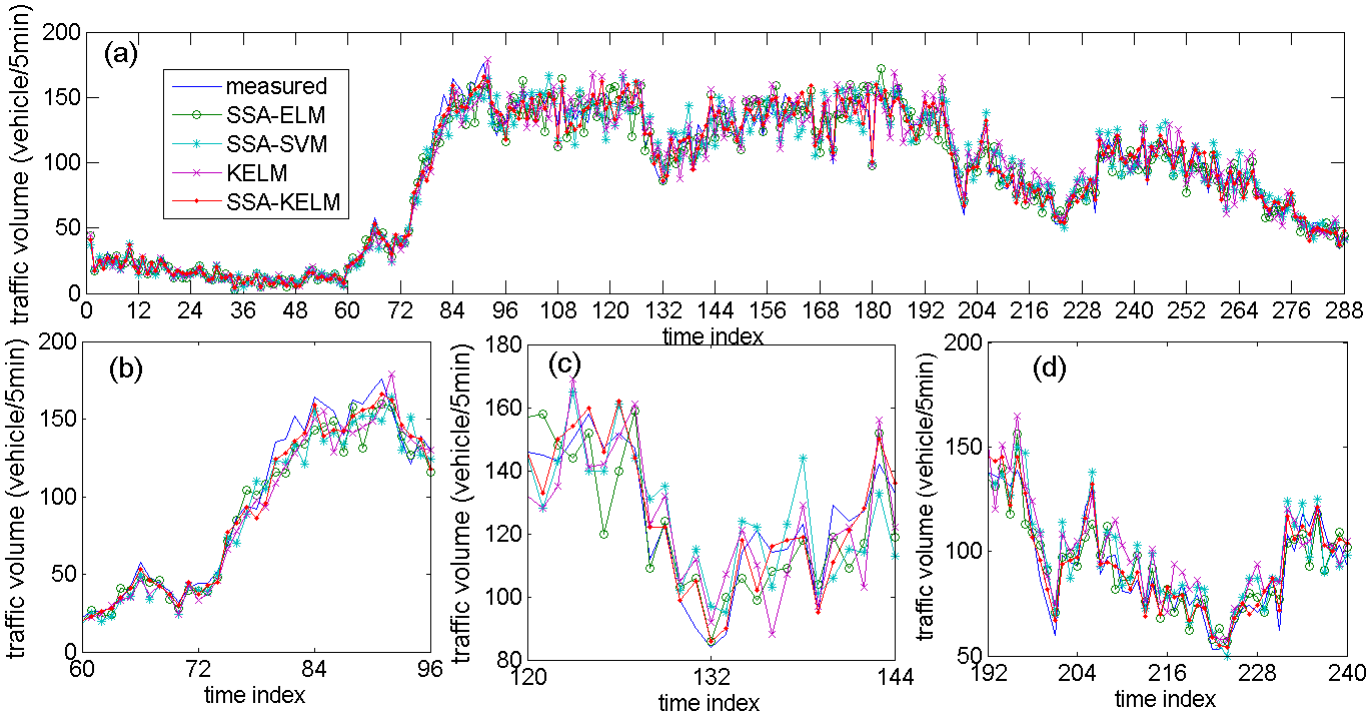
Prediction results of related models based on NO. DC00004965 detector’s data (a) and its parts specific prediction results (b), (c), (d).

**Fig 14:**
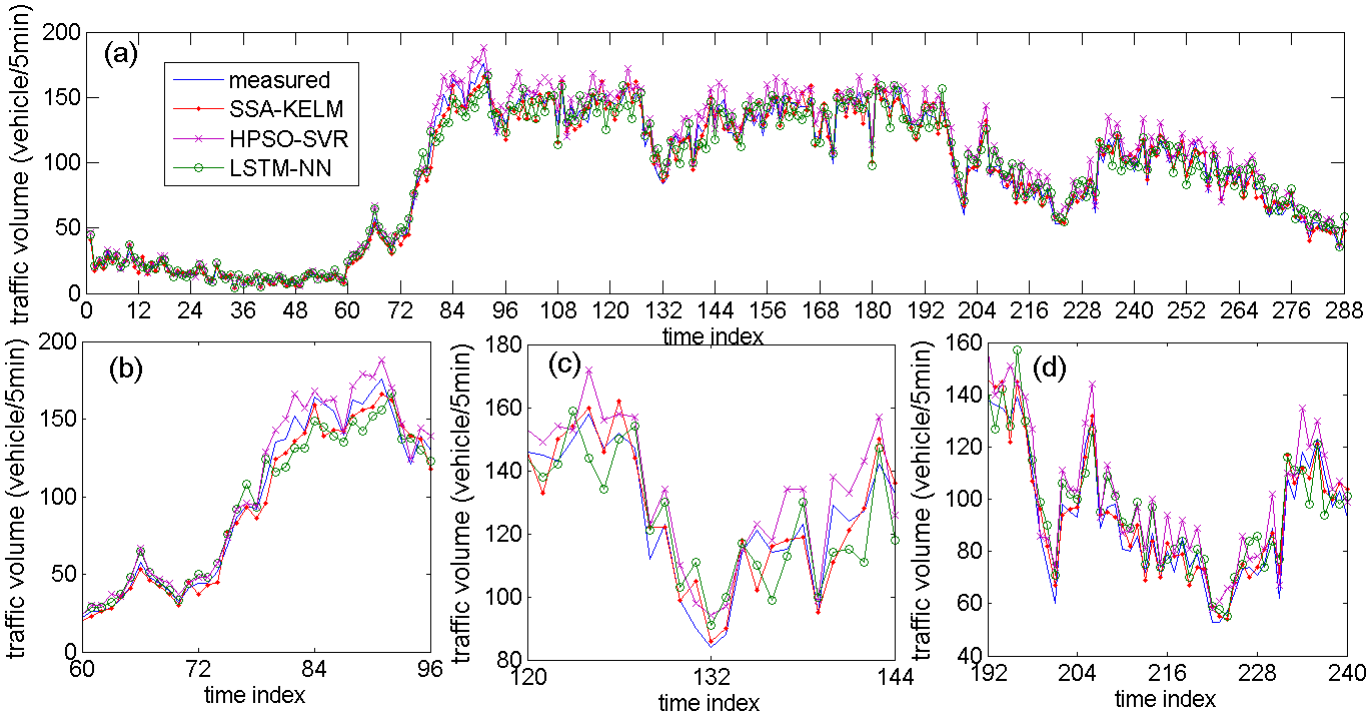
Prediction results of state-of-the-art models based on NO. DC00004965 detector’s data (a) and its parts specific prediction results (b), (c), (d).

**Fig 15:**
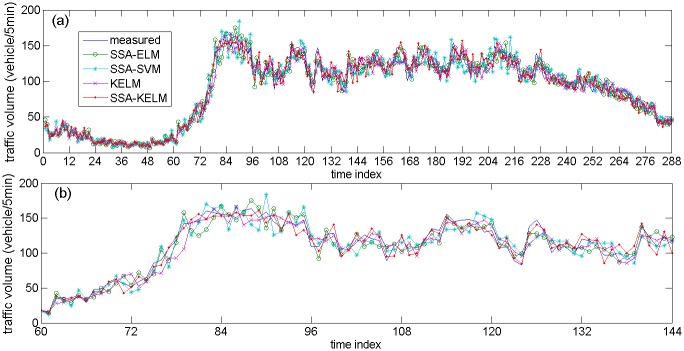
Prediction results of related models based on NO. DC00004966 detector’s data (a) and its parts specific prediction results (b).

**Fig 16:**
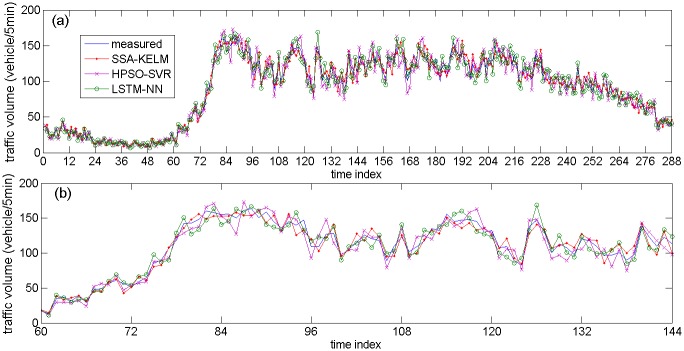
Prediction results of state-of-the-art models based on NO. DC00004966 detector’s data (a) and its parts specific prediction results (b).

**Fig 17:**
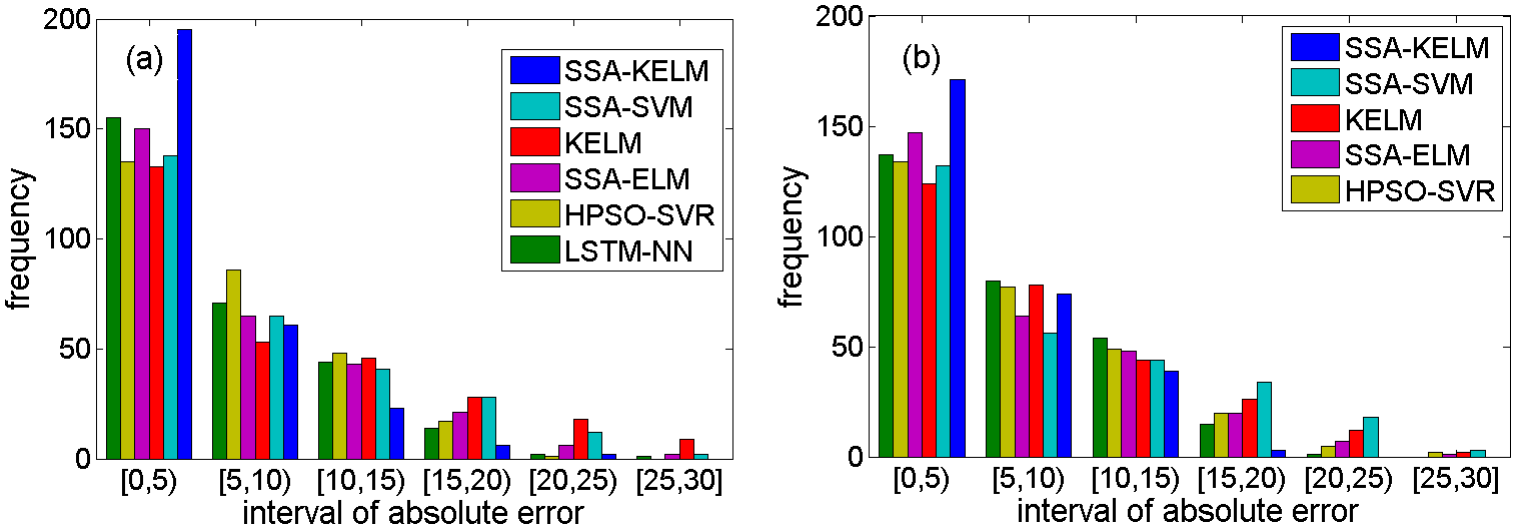
The frequency of absolute error distribution for all models using (a) NO. DC00004965 detector’s data and (b) NO. DC00004966 detector’s data.

Fig 13(a) describes the prediction results of the related models based on NO. DC00004965 detector’s data, Fig 13(b), 13(c) and 13(d) are specific parts of the prediction results. Fig 14(a) describes the prediction results of the state-of-the-art models based on NO. DC00004965 detector’s data. Fig 14(b), 14(c) and 14(d) are specific parts of the prediction results. Fig 15(a) describes the prediction results of related models based on NO. DC00004966 detector’s data. Fig 15(b) is a specific part of the comparison results. Fig 16(a) describes the prediction results of state-of-the-art models based on NO. DC00004966 detector’s data. Fig 16(b) is a specific part of the comparison results. As shown in Figs 13(a), 14(a), 15(a) and 16(a), it is clearly that the SSA-KELM model have the best fitting performance, especially when the traffic volume changes greatly (it is clearly shown in Figs 13(b), 13(c), 13(d), 14(b), 14(c), 14(d), 15(b) and 16(b)). Fig 17(a) and 17(b) present the frequency of absolute error distribution for all models based on NO. DC00004965 detector’s data and NO. DC00004966 detector’s data, respectively. For the SSA-KELM model, we can clearly see that frequency distribution of absolute errors in the interval [0,5) is the highest, and almost all the absolute errors of SSA-KELM model are less than 25, which illustrates the prediction model has better stability.

Table 3 gives the prediction accuracy by using two statistical indices (MAE and MAPE). The prediction accuracy of the SSA-KELM model is improved significantly compared with the other three models. More precisely, in the aspect of MAE, the SSA-KELM model gets an extra 28.13% improvement over the SSA-ELM model, an extra 38.53% improvement over the SSA-SVM model, an extra 40.46% improvement over the KELM model, an extra 26.08% improvement over the HPSO-SVR model, and an extra 22.94% improvement over the LSTM-NN model. In the aspect of MAPE, the SSA-KELM model gets an extra 27.55% improvement over the SSA-ELM model, an extra 36.58% improvement over the SSA-SVM model, an extra 38.32% improvement over the KELM model, an extra 29.37% improvement over the HPSO-SVR model, and an extra 24.62% improvement over the LSTM-NN model. The traffic volume time series shown in Figs 13(b), 13(c), 13(d) and 15(b) are denoted as subcase 1, 2, 3 and 4 respectively, because of their great changing during the period (include traffic state transitional period). The performance indices for these four subcases are shown in Table 4, which illustrate that performance of the SSA-KELM model is also better than the other models, when traffic volume changes greatly.

**Table 3:** Performance indices for different models.

Model	Detector (DC00004965)		Detector (DC00004966)		Mean of the two detectors	
	MAE	MAPE	MAE	MAPE	MAE	MAPE
**SSA-ELM**	6.78	8.80%	6.93	8.61%	6.86	8.71%
**SSA-SVM**	7.66	10.00%	8.38	9.89%	8.02	9.95%
**KELM**	8.64	10.95%	7.92	9.51%	8.28	10.23%
**SSA-KELM**	4.68	6.44%	5.18	6.18%	4.93	6.31%
**HPSO-SVR**	6.82	8.98%	7.13	8.36%	6.98	8.67%
**LSRM-RNN**	6.34	8.54%	6.73	8.02%	6.54	8.28%

**Table 4:** Performance indices of different models for the four subcases.

Model	MAE				MAPE			
	Subcase 1	Subcase 2	Subcase 3	Subcase 4	Subcase 1	Subcase 2	Subcase 3	Subcase 4
**SSA-ELM**	8.16	9.8	8.04	8.98	8.91%	7.92%	8.92%	8.96%
**SSA-SVM**	9.92	11.20	8.76	10.84	11.05%	9.35%	10.03%	10.20%
**KELM**	9.78	11.64	10.69	9.65	10.56%	9.64%	12.17%	9.80%
**SSA-KELM**	6.30	5.36	5.00	6.59	6.62%	4.37%	5.71%	6.41%
**HPSO-SVR**	6.86	8.84	8.71	8.65	8.54%	7.92%	9.75%	8.39%
**LSTM-RNN**	8.78	8.72	7.45	8.08	8.97%	7.34%	8.59%	7.56%

Comparison results of the SSA-KELM model, the SSA-ELM model and the SSA-SVM model show that the performance of KELM is better than ELM and SVM in this case. Comparison results of the SSA-KELM model and the KELM model show that using SSA to filter noise of the original traffic volume time series can improve the model’s performance effectively. Moreover, the experimental results also demonstrate that the SSA-KELM model achieves good prediction performance using both NO. DC00004965 detector’s data and NO. DC00004966 detector’s data, which illustrate that SSA-KELM model has a robust generalization ability. Comparison results of the SSA-KELM model, HPSO-SVR model and LSTM-NN model show that the SSA-KELM model can further improve the accuracy of short-term traffic flow prediction.

### 3.7 Non-parametric tests for multiple comparisons of different models

In order to find significant differences among the results obtained by the studied models, statistical analysis should be performed. In this paper, non-parametric tests are used as suggested in Refs. [59–61]. The parametric statistical analysis loses its credibility because the initial conditions guaranteeing its reliability may not be satisfied [59]. They can offer safe and reliable procedures to contrast the differences between different techniques, especially in multiple-problem analysis.

To compare the performance of multiple models on the multiple data sets, Friedman aligned-ranks test is conducted according to the suggestions of Ref. [60]. Table 5 shows the average ranking of all models. In terms of both MAE and MAPE, the performances of all models can be sorted by average ranking into the following order: SSA-KELM, LSTM-NN, HPSO-SVR, SSA-ELM, SSA-SVM and KELM. It means that SSA-KELM and KELM are the best and worst ones among the six models, respectively. Then, if significant differences are found, we will check whether the control model (the best one) significantly outperformed the others (that is, 1×n comparison) using post hoc tests (such as Holm, Hochberg, Hommel and Finner). In this study, we choose the best performing model, SSA-KELM, as the control model for being compared with the rest of models. Under the null hypothesis, the two models are equivalent. If the null hypothesis is rejected, then the performances of these two models are significantly different. In this paper, we only discuss whether the hypotheses are rejected at the 0.05 level of significance. Tables 6 and 7 show the results of adjusted *p* values in terms of MAE and MAPE respectively, where the *p* values below 0.05 are shown in bold. In terms of MAE, Holm, Hochberg and Hommel tests reject hypotheses 1–3, while Finnner test rejects hypotheses 1–4. In terms of MAPE, all the post hoc tests reject hypotheses 1–4. All the results of post hoc tests show that the performance of SSA-KELM is significantly better than that of SSA-ELM, SSA-SVM and EKLM. Partial results of post hoc tests show that the performance of SSA-KELM is significantly better than that of HPSO-SVR. Though SSA-KELM is not statistically better than LSTM-NN, SSA-KELM outperforms LSTM-NN according to the results of the average ranking.

**Table 5:** Average Rankings of the models using Aligned Friedman.

Model	Ranking (MAE)	Ranking (MAPE)
SSA-KELM	3.5	3.5
KELM	31.3333	31.3333
SSA-SVM	29.6667	29.6667
SSA-ELM	18.3333	18.0833
HPSO-SVR	16	17.5833
LSTM-NN	12.1667	10.8333

**Table 6:** Adjusted *p*-values of the models in terms of MAE.

i	Model	unadjusted *p*	*p*_Holm_	*p*_Hochberg_	*p*_Hommel_	*p*_Finner_
1	KELM	0.000005	**0.000024**	**0.000024**	**0.000024**	**0.000024**
2	SSA-SVM	0.000017	**0.000068**	**0.000068**	**0.000068**	**0.000042**
3	SSA-ELM	0.014745	**0.044235**	**0.044235**	**0.044235**	**0.024454**
4	HPSO-SVR	0.03988	0.07976	0.07976	0.07976	**0.049599**
5	LSTM-NN	0.154218	0.154218	0.154218	0.154218	0.154218

**Table 7:** Adjusted *p*-values of the models in terms of MAPE.

i	Model	unadjusted *p*	*p*_Holm_	*p*_Hochberg_	*p*_Hommel_	*p*_Finner_
1	KELM	0.000005	**0.000024**	**0.000024**	**0.000024**	**0.000024**
2	SSA-SVM	0.000017	**0.000068**	**0.000068**	**0.000068**	**0.000042**
3	SSA-ELM	0.016508	**0.049524**	**0.041195**	**0.033016**	**0.027362**
4	HPSO-SVR	0.020597	**0.049524**	**0.041195**	**0.041195**	**0.027362**
5	LSTM-NN	0.227975	0.227975	0.227975	0.227975	0.227975

## 4 Conclusions

A novel short-term traffic flow prediction model (SSA-KELM) is proposed based on SSA and KELM. The prediction accuracy of SSA-KELM model compares with that of SSA-ELM model, SSA-SVM model and single KELM model using the same real-world traffic volume data. The prediction results are encouraging, especially at the period that traffic volume changes greatly. The main contributions of this paper are not only the introduction of KELM method for traffic flow prediction and how to optimize the model parameters based on GSA, but also considering the chaotic characteristics of short-term traffic flow and determining the model’s optimal input form based on PSR. It is worth noting that filtering original traffic volume data in the preprocessing stage is important. The new time series reconstructed by SSA retains the main characteristics of the original traffic volume time series (the contribution ratio is 98.27%), but has filtered the main noise components. A conclusion can be got that the proposed model is an effective and accurate method for short-time traffic flow prediction, which can provide satisfactory prediction results. Although the experimental results presented here are promising and the SSA-KELM model can be successfully applied to predict traffic flow, this model suffers from weak interpretability like other ANN-based models. In future work, the model with well interpretability can be introduced into the proposed model, such as fuzzy system and its improvement [62–64]. It is difficult to improve the interpretability of the model while keeping the accuracy of the model. And it is a difficulty that how to reflect the characteristics of traffic flow in the model.

To obtain more general and robust conclusions, traffic flow data from different roadways and more complicated traffic conditions should be used to test the proposed model. Moreover, it is required that the other data sets of traffic flow parameters (such as travel time, average traffic speed and average occupancy, this study chooses the traffic volume as the demonstration) are applied to test the model. Furthermore, traffic flow parameters data set with different time intervals (such as 1 min, 2 min and 10 min) should be used to test the proposed model for further study. Other advanced optimization algorithms should be further studied to search for more appropriate parameter combinations of the model and to obtain more accurate results of short-term traffic flow prediction.

## Supporting Information

S1 Dataset The traffic volume data from the two detectors. (XLSX)
